# IMF deposition ceRNA network analysis and functional study of HIF1a in yak

**DOI:** 10.3389/fvets.2023.1272238

**Published:** 2023-10-17

**Authors:** Mengning Luo, Hui Wang, Jun Zhang, Kangzhu Yixi, Shi Shu, Changqi Fu, Jincheng Zhong, Wei Peng

**Affiliations:** ^1^Key Laboratory of Qinghai-Tibetan Plateau Animal Genetic Resource Reservation and Utilization, Sichuan Province and Ministry of Education, Southwest Minzu University, Chengdu, China; ^2^Qinghai Academy of Animal Science and Veterinary Medicine, Qinghai University, Xining, China

**Keywords:** IMF, adipogenesis, RNA-seq, *HIF1α*, ceRNA, yak

## Abstract

The concentration of intramuscular fat (IMF) is a crucial determinant of yak meat quality. However, the molecular mechanisms that regulate IMF in yak remain largely elusive. In our study, we conducted transcriptome sequencing on the longissimus dorsi muscle tissues of yaks with varying IMF contents. We then filtered differentially expressed genes (DEGs), microRNAs (DEMs), and long non-coding RNAs (DELs) to elucidate potential regulatory pathways of adipogenesis in yaks. Overall, our research sheds light on an array of potential mRNAs and noncoding RNAs implicated in IMF deposition and elaborates on the role of *HIF1α* in yaks. These findings contribute valuable insights that can serve as a guide for further research into the molecular mechanisms governing IMF deposition.

## 1. Introduction

The yak (*Bos grunniens*), a distinctive bovine species native to the Qinghai-Tibet Plateau, is exceptionally adept at withstanding the severe local conditions, such as freezing temperatures and oxygen scarcity. Currently, China’s yak population is estimated at 18 million, comprising roughly 18% of the country’s beef cattle inventory. Yaks have served as a crucial livestock resource for herdsmen, offering bountiful supplies of meat, milk, and fur, and playing a critical role in the socioeconomic structure of the local communities.

With the current trend toward health consciousness, high-quality, sustainable beef has garnered international attention (1, 2). Yet, the acceptability of yak meat is somewhat hampered by its low yield, dense muscle fibers, minimal intramuscular fat (IMF) content, and limited tenderness. The development of muscle and IMF are complex processes that contribute significantly to economic factors and meat quality. These developments begin early in bovine embryogenesis, with the proliferation of myoblast progenitors and mononuclear myoblasts forming multinuclear muscle fibers (3). Fat formation commences during the second trimester (4), stemming from the proliferation and differentiation of intramuscular preadipocytes. Thus, unraveling the genetic regulation mechanisms behind IMF formation and muscle development is of paramount importance to enhancing yak meat quality.

The content of IMF significantly influences beef’s tenderness and juiciness, making its optimization a focal point of research (5). Various studies have identified numerous lipid metabolism-associated genes linked with IMF deposition, including adiponectin, C1Q and collagen domain containing (ADIPOQ) (6), peroxisome proliferator-activated receptor gamma (PPARγ or PPARG) (7), thyroid hormone responsive (8), and fatty acid-binding protein 4 (FABP4) (9). A genome-wide association study proposed that PPARG coactivator 1 alpha, hepatocyte nuclear factor 4 gamma, and forkhead box P3 are implicated in IMF deposition in cattle (9). Comparative transcriptome analysis identified Phosphoenolpyruvate Carboxykinase 1 (PCK1) as significantly associated with IMF (10). Our prior work identified several key genes involved in IMF deposition in yaks, including *LPL* (11), *ACADL* (11), *SCD* (11), *FASN* (11), lncFAM200B (12), and miR-6529a (12). Additionally, ChIP-seq and mRNA-seq showed that SIRT1 plays an epigenetic role in IMF deposition through H3K4ac in yaks (13). Despite numerous studies, the molecular mechanisms regulating IMF deposition in yaks remain elusive.

This study aims to identify and scrutinize functional genes related to IMF development in yak longissimus dorsi muscle. By combining analyses, we constructed a competitive endogenous RNA (ceRNA) regulatory network, through which we identified several potential miRNA-lncRNA-mRNA regulatory axes possibly related to IMF deposition. Finally, we confirmed the regulatory effect of a candidate gene, *HIF1α*, on the proliferation and differentiation of yak intramuscular preadipocytes.

## 2. Materials and methods

### 2.1. Ethics statement

This research was endorsed by the Committee for Animal Ethics of the College of Animal Science and Technology, Southwest Minzu University (Permit number: SMU 202106010). We ensured all experimental protocols complied with the guidelines set by the approving committee. We obtained written permission from the owner of the yak to take samples.

### 2.2. Sample collection

Samples were collected from Xiaojin County, located in Sichuan Province, China. Following the slaughter of yaks, longissimus dorsi tissues from 24 yaks were sterilely dissected and preserved in liquid nitrogen. The IMF content of each sample was ascertained following the standard Soxhlet extraction method. Subsequently, six samples - three highest IMF content (4.50 ± 0.20) (H-IMF) and three lowest IMF content (2.52 ± 0.11) (L-IMF) - were selected from 24 (3.50 ± 0.68) samples for total RNA extraction and sequencing.

### 2.3. Total RNA isolation, sequencing and data processing

The total RNA was extracted by TRIzol (invitrogen, Virginia, USA) reagent according to the manufacturer’s instructions. The extracted RNA was used for the lncRNA and miRNA library construction according to previous standard method. The RNA libraries (lncRNA and miRNA) were then sequenced by the Illumina HiSeqTM 4,000 (Gene *Denovo*, Guangzhou, China) platform. After completing the sequencing of the lncRNA library, we obtained data about both mRNA and lncRNA.

For lncRNA and mRNA sequencing results, quality control of raw reads was conducted using Fastp (V0.18.0) (14). We removed reads containing adaptors and unknown nucleotides (N) greater than 10%, as well as all low-quality reads with an overabundance of adenine bases (A) (> 50% base, *Q* value ≤20). This process resulted in high-quality clean reads, which served as the basis for all downstream analyses. Initially, Bowtie2 (V2.2.8) (15) was employed to align the clean reads to the yak ribosome database. Following the exclusion of the ribosomal reads, the remaining unmapped reads were used for subsequent transcriptome analysis. Next, we utilized HISAT2 (V2.1.0) (16) to conduct a comparative analysis based on the reference genome (Ensembl_release100), and the transcripts were reconstructed with StringTie (V1.3.4). Finally, the differential expression patterns of lncRNAs and mRNAs were analyzed via the Gene Denovo platform. We normalized the expression levels of lncRNA and mRNA using the FPKM (fragments per kilobase of transcript per million mapped reads) method.

For miRNA analysis, we performed a series of filtering steps on the raw reads. These included removing reads containing 5′ adaptors, low-quality reads (those with more than one base having a quality value of less than 20 or reads containing unidentified nucleotides), reads without insert fragments, reads with inserted fragments shorter than 18 nucleotides, and reads containing predominantly adenine bases (more than 70% of the bases in a read are A). These steps helped us secure high-quality reads. After removing duplicate reads, we aligned all unannotated tags with the reference genome and identified potential novel miRNA candidates. We calculated miRNA expression levels using transcripts per million (TPM) as a measure. The formula for TPM is as follows: TPM = T * 10^6 / N, where T represents the tags of miRNA and N denotes the total miRNA tags (the sum of existing, existing editing, known, and newly identified miRNA counts).

### 2.4. Analysis of differentially expressed lncRNAs, mRNAs and miRNAs

DESeq2 (17) software was utilized to analyze the significance of lncRNAs and mRNAs, while the edgeR (18) package was employed for miRNA screening. The selection criteria for differentially expressed lncRNA, mRNA and miRNA were set as an expression level change of more than 1.5-fold, with a *p* value ≤0.05 between the H-IMF and L-IMF groups.

### 2.5. Prediction of target genes and functional enrichment analysis

The prediction of differentially expressed miRNAs (DEMs) targets was performed using miRanda (v3.3a) and TargetScan (Version:7.0) software. The common genes predicted by both databases were established as the final target genes for each DEM. Target prediction for differentially expressed lncRNAs (DELs) utilized three strategies: antisense, cis-acting and trans-acting regulation prediction. For antisense regulation, RNAplex software identified targets by comparing complementary bases between the lncRNA and mRNA. For cis-acting prediction, gene locations within a 10 kb radius of the lncRNA on the yak genome were identified as cis-acting regulatory targets. For trans-acting targets, co-expression of lncRNA and mRNA, regardless of mRNA location, was a key determinant. LncTar was used to calculate free energy between lncRNA and mRNA to predict lncRNA targets.

The potential biological functions and regulatory pathways of differentially expressed mRNAs (DEGs), DEM targets and DEL targets were analyzed using Gene Ontology (GO) and Kyoto Encyclopedia of Genes and Genomes (KEGG) enrichment analyses, conducted with the R package ClusterProfiler (3.8.1) (19). Statistical significance was assigned at a *p* value ≤0.05.

### 2.6. Construction of the ceRNA network

We calculated the Spearman rank correlation coefficient between miRNA and candidate ceRNAs (lncRNAs and mRNAs). We selected target gene pairs with a correlation coefficient of ≤ −0.7. Pearson correlation coefficients between ceRNA pairs were also calculated. Pairs with a correlation coefficient greater than 0.9 were considered as potential ceRNA pairs. Based on these results, we performed a hypergeometric cumulative distribution function test. ceRNA pairs with a *p* value of ≤0.05 were chosen as the final ceRNA pairs. Finally, using Cytoscape software, we constructed a ceRNA regulation map.

### 2.7. RT-qPCR validation

The relative expression levels of genes were determined using the TB Green Premix EX TaqTM II kit (TaKaRa, Osaka, Japan) through quantitative reverse-transcription polymerase chain reaction (RT-qPCR). Primer information is provided in Supplementary Table S1. We chose the *β–actin* gene as the normalization control for mRNAs and lncRNAs, and the *U6* gene as the normalization control for miRNAs, and relative expression levels were computed using the 2-ΔΔCt method.

### 2.8. Construction of adenovirus and siRNA synthesis

Recombinant adenoviruses were created following a previously established procedure. Briefly, the cDNA of *HIF1α* from yak was amplified and cloned into the pAV[Exp]-GFP-CMV vector (named Ad-*HIF1α*). An adenoviral plasmid containing GFP (named Ad-*GFP*) served as the control. The small interfering RNA (siRNA) for *HIF1α* (sense GCCGCUCAAUUUAUGAAUATT, antisense UAUUCAUA AAUUGAGCGGCTT, named si-*HIF1α*) and a negative control (sense UUCUCCGAACGUGUCACGUTT, antisense ACGUGACACG UUCGGAGAATT, named NC) were synthesized by Sigma (USA).

### 2.9. Cell culture and transfection

Cells were cultured in DMEM/F12 medium (Hyclone, USA), supplemented with 10% fetal bovine serum (Hyclone) and 1% penicillin–streptomycin (Boster, Switzerland), and incubated at 37°C with 5% CO_2_. Upon reaching 80% confluence, cells were serum-starved for 4 h before siRNA transfection, carried out using LipofectamineTM3000 reagent (Invitrogen) as per the manufacturer’s instructions. Adenovirus was injected according to the formula: volume (μL) = MOI (1000) * number of cells / adenovirus titers (IFU/ml) * 1000. After transfection for 6 h, the medium was replaced with induction medium (complete medium containing oleic acid), supplemented with 200 μM cobalt (II) chloride hexahydrate (Sigma). Cells were harvested 48 h later for subsequent experiments.

### 2.10. Oil Red O staining and intracellular triglyceride determination

Adherent cells in a 12-well plate were first gently washed with PBS solution. Subsequently, 400 μL of 4% paraformaldehyde (Biosharp, China) was added to fix the cells for 1 h at 20°C. Afterward, the fixative was removed by washing with PBS solution, and the cells were stained with Oil Red O reagent (Sigma). This reagent was prepared by dissolving it in isopropanol solution and diluted with PBS at a 3:2 ratio. After staining the fixed cells for 30 min at 20°C, they were washed five times with PBS solution. The cells were then observed and photographed. Oil Red O dye was extracted with isopropyl alcohol and OD values were determined. The intracellular triglyceride (TG) levels were determined using a triglyceride assay kit (Applygen Technologies Inc., China) as per the manufacturer’s instructions. Briefly, the total proteins were first extracted and detected using RIPI reagent (Beyotime, Shanghai, China) and a Pierce BCA protein assay kit (Thermo Fisher Scientific, Massachusetts, USA), respectively. The intracellular TG was then detected and the content was normalized to the total protein content.

### 2.11. CCK-8 assay

The CCK-8 assay was employed to analyze cell proliferation. Cells were inoculated in a 96-well plate and cultured in fresh complete medium for 0, 24, 48, 72, and 96 h, respectively. Ten microliters of CCK-8 reagent was added to each well and incubated for 2 h at 37°C. Absorbance was measured at 480 nm wavelength after returning to room temperature.

### 2.12. Statistical analysis

One-way ANOVA was performed using SPSS (version 25.0; IBM Corp, Armonk, NY, USA), and the significance was determined using Duncan’s test. Significance was reported at *p* ≤ 0.05. The data are presented as Mean ± SD (standard deviation) from three biological replicates and three technical replicates. Indicators *** signify *p* < 0.001, ** denote *p* < 0.01, and * represent *p* < 0.05.

## 3. Results

### 3.1. Overview of sequencing results

**Figure 1:**
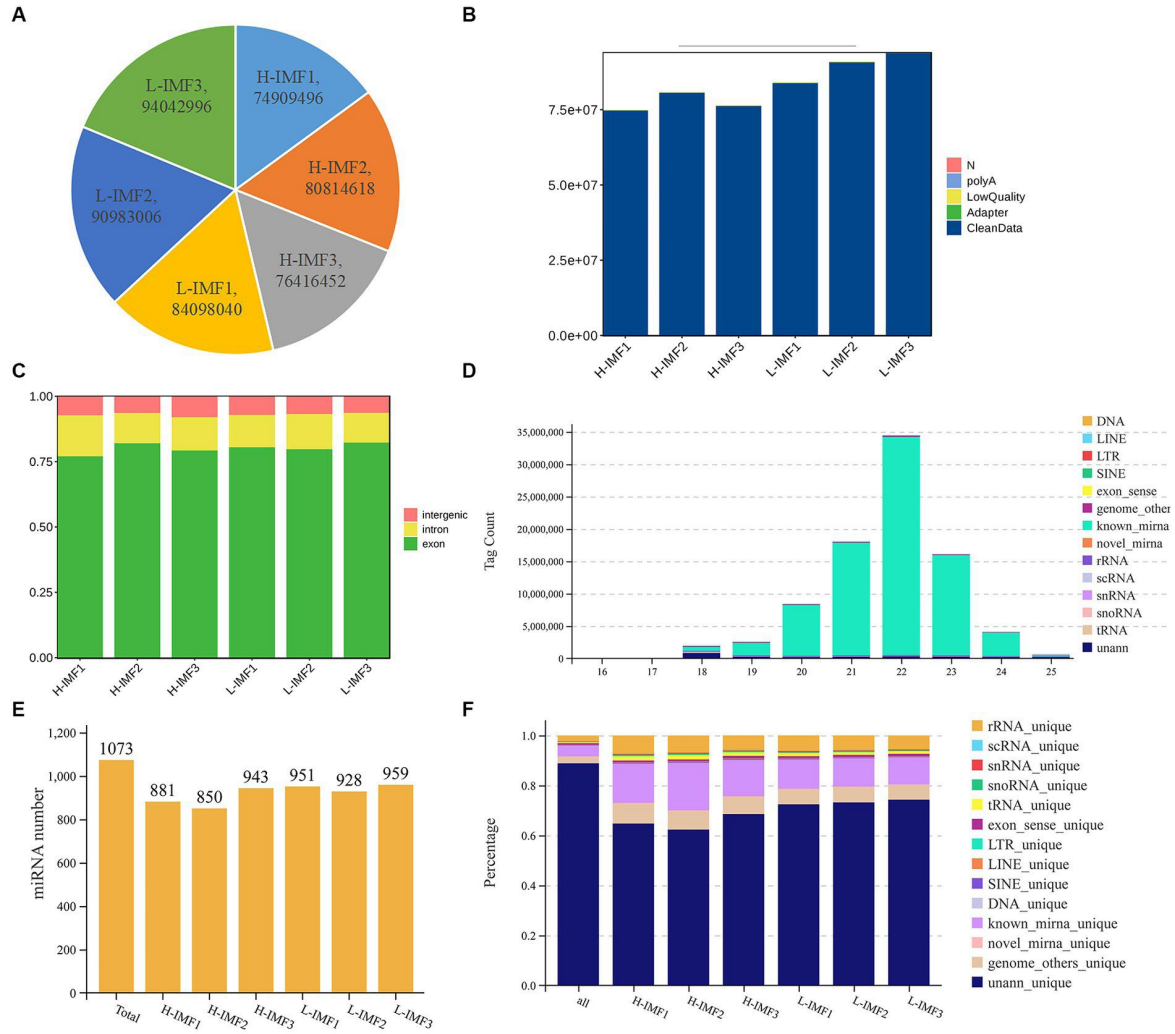
Quality control of lncRNA and miRNA. **(A)** Raw reads statistics. **(B)** Statistics after filtering. **(C)** Comparative statistical map of reference regions. **(D)** RNA length statistics. **(E)** Total identified miRNA statistics. **(F)** RNA alignment statistics.

The lncRNA sequencing results yielded an average of 773,801,889 raw reads for the H-IMF group and 89,708,014 raw reads for the L-IMF group (Figure 1A; Supplementary Table S2). Following quality control, an average of 77,201,181 (99.77%) and 89,469,916 (99.73%) clean reads were obtained for the respective groups (Figure 1B; Supplementary Table S2). Approximately 91.29 to 92.85% of the clean reads were successfully mapped to the yak reference genome. Out of these, 73.33 to 76.47% of the reads were uniquely mapped to specific regions of the genome, while 15.83 to 17.99% showed multiple genomic locations (Supplementary Table S2). These findings demonstrate the reliability of the sequencing data obtained from the longissimus dorsi muscle of yak, making it suitable for subsequent bioinformatics analysis. The even distribution of a large number of reads across the genes indicates high sample quality. Furthermore, the ratio of reads corresponding to exons, introns, and intergenic regions varied, with exons exhibiting the highest ratio and intergenic regions showing the lowest (Figure 1C). These results suggest that the RNA expression profiles differ among the various samples, indicating specific gene regulation patterns.

For the miRNA sequencing results, we generated a total of 980,758 and 187,527,38 clean reads from the longissimus dorsi muscle of yak in the H-IMF and L-IMF groups, respectively (Supplementary Table S3). Following the removal of low-quality reads and adapter sequences, we obtained 936,603,2 and 162,650,62 clean tags for further analysis (Supplementary Table S3). Of these, 156,302 and 255,847 clean tags were mapped to the yak reference genome, respectively. Tags mapped to exons, introns, and repetitive sequences were excluded (Figure 1F; Supplementary Table S3). Most of the small RNA ranged from 18 to 25 nt, with 22 nt being the most common length (Figure 1D). We identified a total of 1,073 miRNAs for subsequent analysis (Figure 1E).

### 3.2. Differential expression analysis of mRNAs

**Figure 2:**
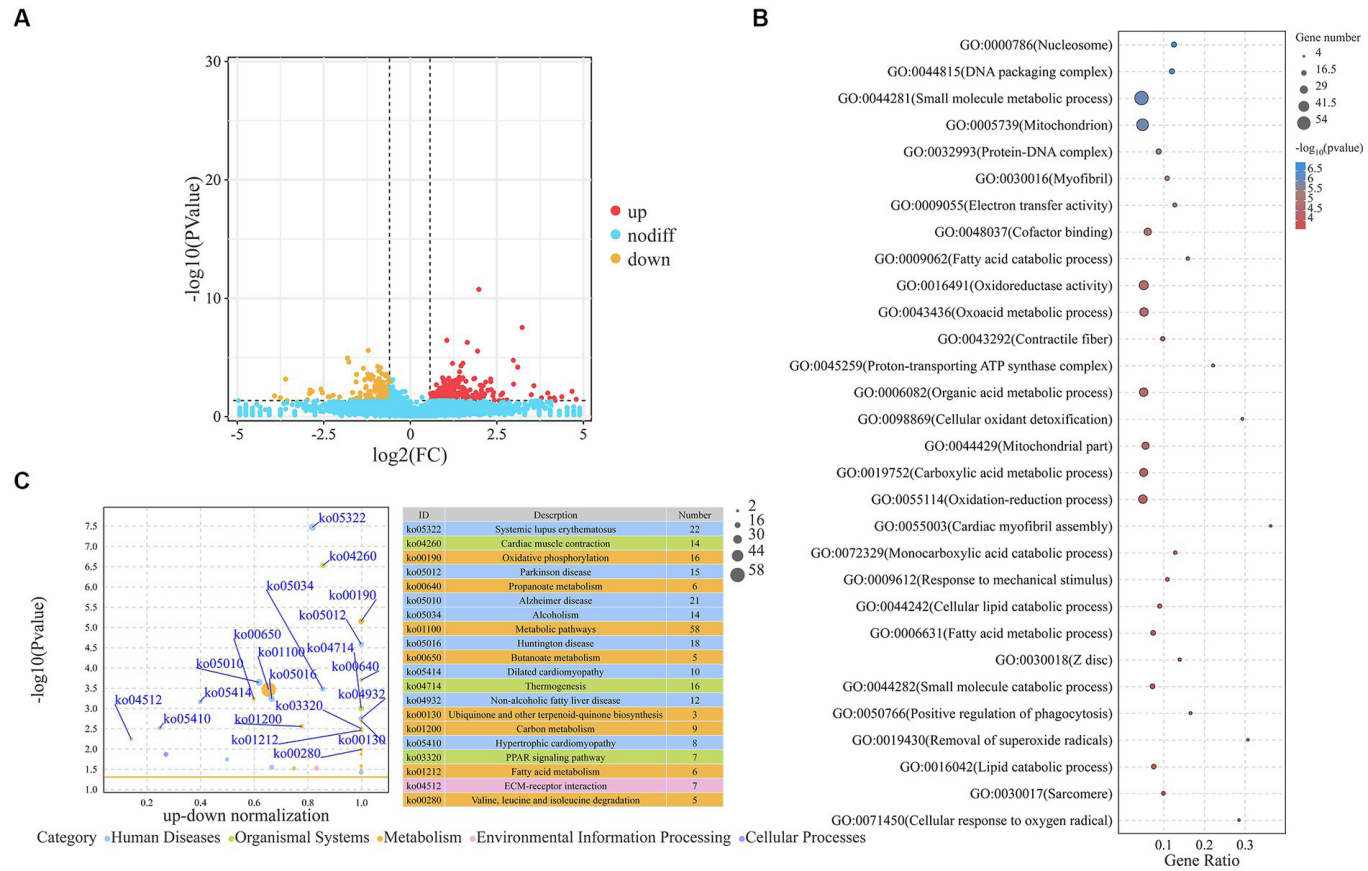
Analysis of DEGs. **(A)** Volcano plot representing DEGs. **(B)** Top 30 GO terms enriched by DEGs. **(C)** Top 20 KEGG pathways associated with DEGs.

We screened the DEGs between the H-IMF and L-IMF groups, yielding 469 DEGs, with 318 upregulated and 151 downregulated (Figure 2A; Supplementary Table S4). These included lipid metabolism-related genes such as galactosidase beta 1 like 2 (*GLB1L2*, log2FC = −2.36), APC regulator of WNT signaling pathway 2 (*APC2*, log2FC = −1.21), heart Fatty acid-binding protein (*H-FABP*, log2FC = 2), phospholipid phosphatase related 2 (*PLPPR2*, log2FC = 1.88), and ELOVL fatty acid elongase 6 (*ELOVL6*, log2FC = 1.64). Muscle development-related genes were also included, such as methyltransferase like 21C (*METTL21C*, log2FC = −2.56), myosin light chain 3 (*MYL3*, log2FC = 3.12), and myosin IG (*MYO1G*, log2FC = 1.06) (Figure 2B; Supplementary Table S5). GO analysis revealed that these DEGs were enriched in lipid metabolism terms, such as regulation of the MAPK cascade (GO:0043408), fatty acid metabolic process (GO:0006631), lipid catabolic process (GO:0016042), fatty acid catabolic process (GO:0009062), lipid oxidation (GO:0034440), fatty acid derivative metabolic process (GO:1901568), and Wnt-protein binding (GO:0017147), as well as muscle development terms, including muscle fiber development (GO:0048747), striated muscle cell development (GO:0055002), muscle tissue development (GO:0060537), and muscle cell development (GO:0055001) (Supplementary Table S6). KEGG analysis revealed that DEGs were significantly annotated in lipid metabolism-related pathways such as metabolic pathways (ko01100), fatty acid metabolism (ko01212), PPAR signaling pathway (ko03320), valine, leucine and isoleucine degradation (ko00280), and fatty acid degradation (ko00071). They were also annotated in muscle development-related pathways, such as cardiac muscle contraction (ko04260) (Figure 2C; Supplementary Table S7).

### 3.3. Differential expression analysis of miRNAs

**Figure 3:**
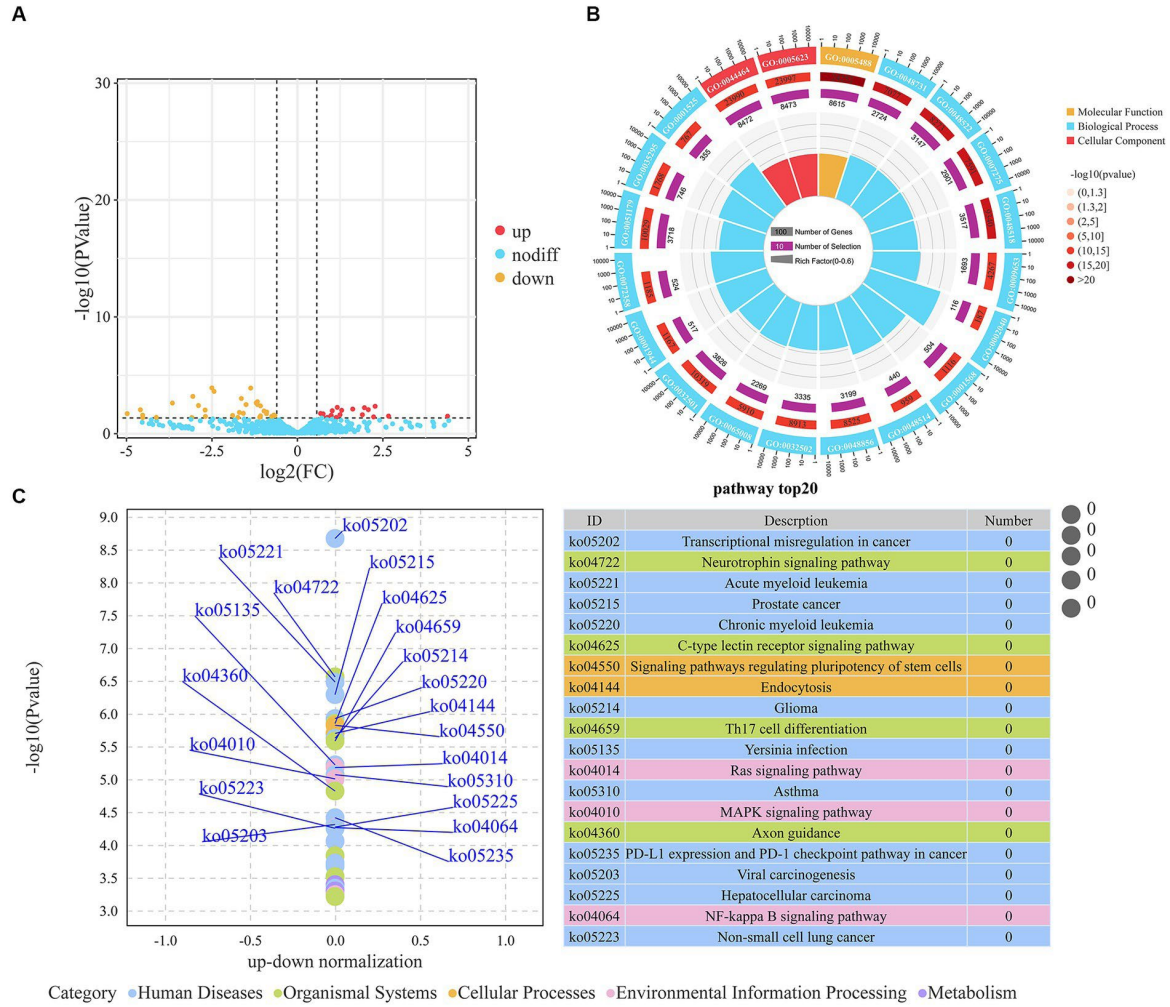
Analysis of DEMs. **(A)** Volcano plot representing DEMs. **(B)** Top 20 GO terms enriched by DEMs. **(C)** Top 20 KEGG pathways associated with DEMs.

We identified a total of 57 DEMs between the H-IMF and L-IMF groups, with 20 upregulated and 37 downregulated (Figure 3A; Supplementary Table S8). We then predicted the targets of these DEMs (Supplementary Table S9) and performed GO and KEGG analyses. GO analysis indicated that the targets of the DEMs were significantly enriched in lipid metabolism-related terms, including ATP binding (GO:0005524), fatty acid biosynthetic process (GO:0006633), phosphatidylinositol binding (GO:0035091), saturated fatty acid elongation (GO:0019367), and very long-chain fatty acid biosynthetic process (GO:0042761) (Figure 3B; Supplementary Table S10).

KEGG pathway analysis showed that the targets of the DEMs were enriched in several signaling and metabolic pathways. These included the MAPK signaling pathway (ko04010), PI3K-Akt signaling pathway (ko04151), FoxO signaling pathway (ko04068), adipocytokine signaling pathway (ko04920), fatty acid metabolism (ko01212), and HIF-1 signaling pathway (ko04066) (Supplementary Table S11). Additionally, we noted enrichment in pathways related to muscle development, such as the regulation of actin cytoskeleton (ko04810), arrhythmogenic right ventricular cardiomyopathy (ko05412), hypertrophic cardiomyopathy (ko05410), and vascular smooth muscle contraction (ko04270) (Figure 3C; Supplementary Table S11).

### 3.4. Differential expression analysis of lncRNAs

**Figure 4:**
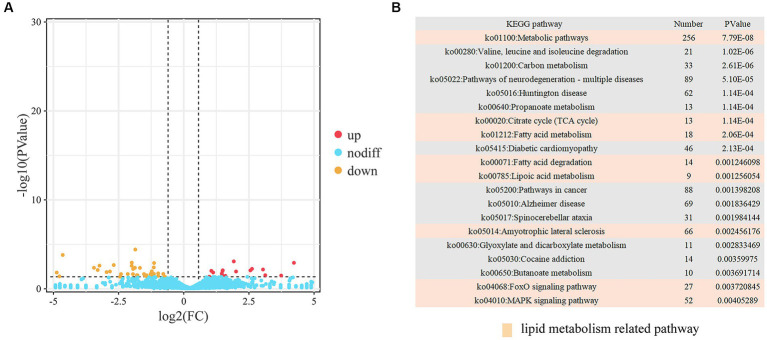
Analysis of DELs. **(A)** Volcano plot representing DELs. **(B)** Top 20 KEGG pathways associated with trans DELs.

We identified a total of 73 DELs, including 25 upregulated and 48 downregulated (Figure 4A; Supplementary Table S12). The targets of these lncRNAs were predicted using three independent algorithms, antisense, cis, and trans. From this analysis, 3,762 target genes were identified. Specifically, 10 genes were found to be targets of 11 antisense lncRNAs, 47 genes were targeted by 45 cis-acting lncRNAs, and 3,705 genes were targeted by 73 trans-acting lncRNAs. Subsequently, we performed KEGG pathway analysis on the identified target genes. The results revealed a significant enrichment in pathways related to both lipid metabolism (such as butanoate metabolism, fatty acid metabolism, PPAR signaling pathway, insulin secretion, and fatty acid degradation) and muscle development (including cardiac muscle contraction, adrenergic signaling in cardiomyocytes, hypertrophic cardiomyopathy, dilated cardiomyopathy, and regulation of actin cytoskeleton) (Figure 4B; Supplementary Table S13).

### 3.5. Construction of the ceRNA network and RT-qPCR validation of gene expression

**Figure 5:**
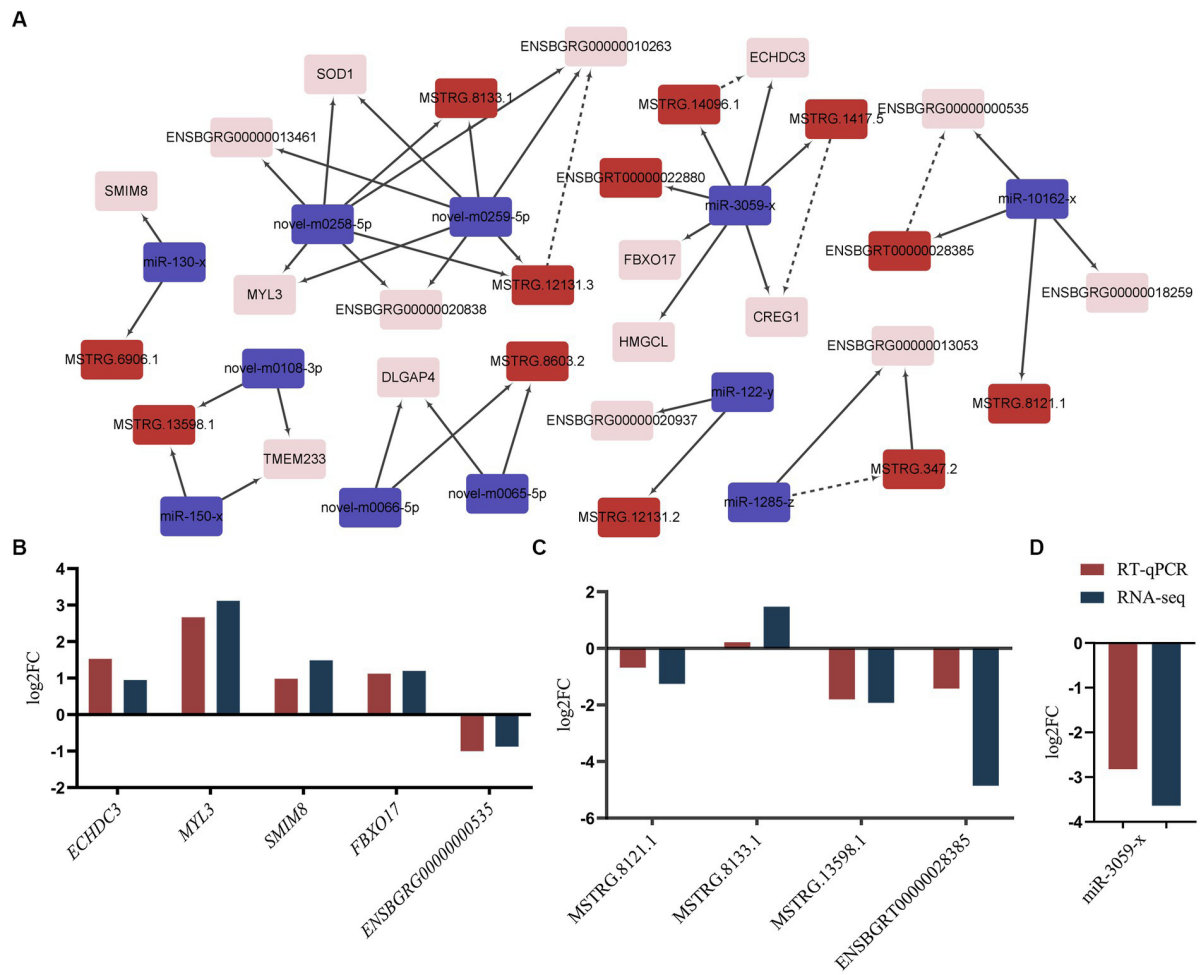
ceRNA co-regulation network and validation. **(A)** ceRNA co-regulation network. Red rectangle: lncRNAs, purple rectangle: miRNAs, pink rectangle: mRNAs. The dotted lines signify co-regulation between lncRNAs and mRNAs, while solid lines represent interactions between miRNAs and other transcripts. **(B–D)** The validation results of mRNAs, lncRNAs and miRNAs. “RNA-seq” denotes the results from RNA-sequencing. “RT-qPCR” represents the results with a log2N transformation. N: Represents the average mRNA expression levels of selected genes as detected by RT-qPCR.

There is mounting evidence indicating that both mRNA and lncRNA can function as ceRNAs for specific miRNAs, playing key roles in regulating gene function across various processes (20). This suggests potential co-regulation of ceRNAs and their corresponding miRNAs in IMF deposition. Integrating the identified lncRNA-mRNA pairs, we constructed a comprehensive ceRNA network. This network encompasses 16 DEGs, 11 DEMs, and 12 DELs, reflecting 26 interaction relationships (Figure 5A). The 16 identified genes were primarily enriched in metabolic pathways (ko01100), valine, leucine, and isoleucine degradation (ko00280), cardiac muscle contraction (ko04260), motor proteins (ko04814), and hypertrophic cardiomyopathy (ko05410) (Supplementary Table S14). Within this network, both MSTRG.12131.3 and its target *ENSBGRG00000010263* were found to be targeted by novel-m0258-5p and novel-m0259-5p. Additionally, *CREG1* and *ECHDC3* are regulated by miR-3059-x. Similar regulatory dynamics were observed in the miR-10162-x-ENSBGRT00000028385-*ENSBGRG00000000535* and miR-1285-z-MSTRG.347.2-*ENSBGRG00000013053* ceRNA sub-networks, implying that *ENSBGRG00000010263*, *ENSBGRG0 0000000535*, *ENSBGRG00000013053*, *CREG1*, and *ECHDC3* could be crucial genes modulated by noncoding RNAs in regulating IMF deposition.

To validate the RNA-Seq results, we performed RT-qPCR on a random selection of RNAs. These included five mRNAs (*SMIM8*, *FBXO17*, *MYL3*, *ECHDC3*, and *ENSBGRG00000000535*) (Figure 5B), four lncRNAs (MSTRG.13598.1, MSTRG.8133.1, MSTRG.8121.1, and ENSBGRT00000028385) (Figure 5C), and one miRNA (miR-3059-x) (Figure 5D). The expression levels of these selected RNAs significantly varied in the longissimus dorsi muscle of yaks with differing fat content. Furthermore, the observed expression patterns were highly congruent with the results acquired via the RNA-Seq approach.

### 3.6. Investigation of *HIF1α*’s effect on yak intramuscular preadipocyte proliferation and differentiation

**Figure 6:**
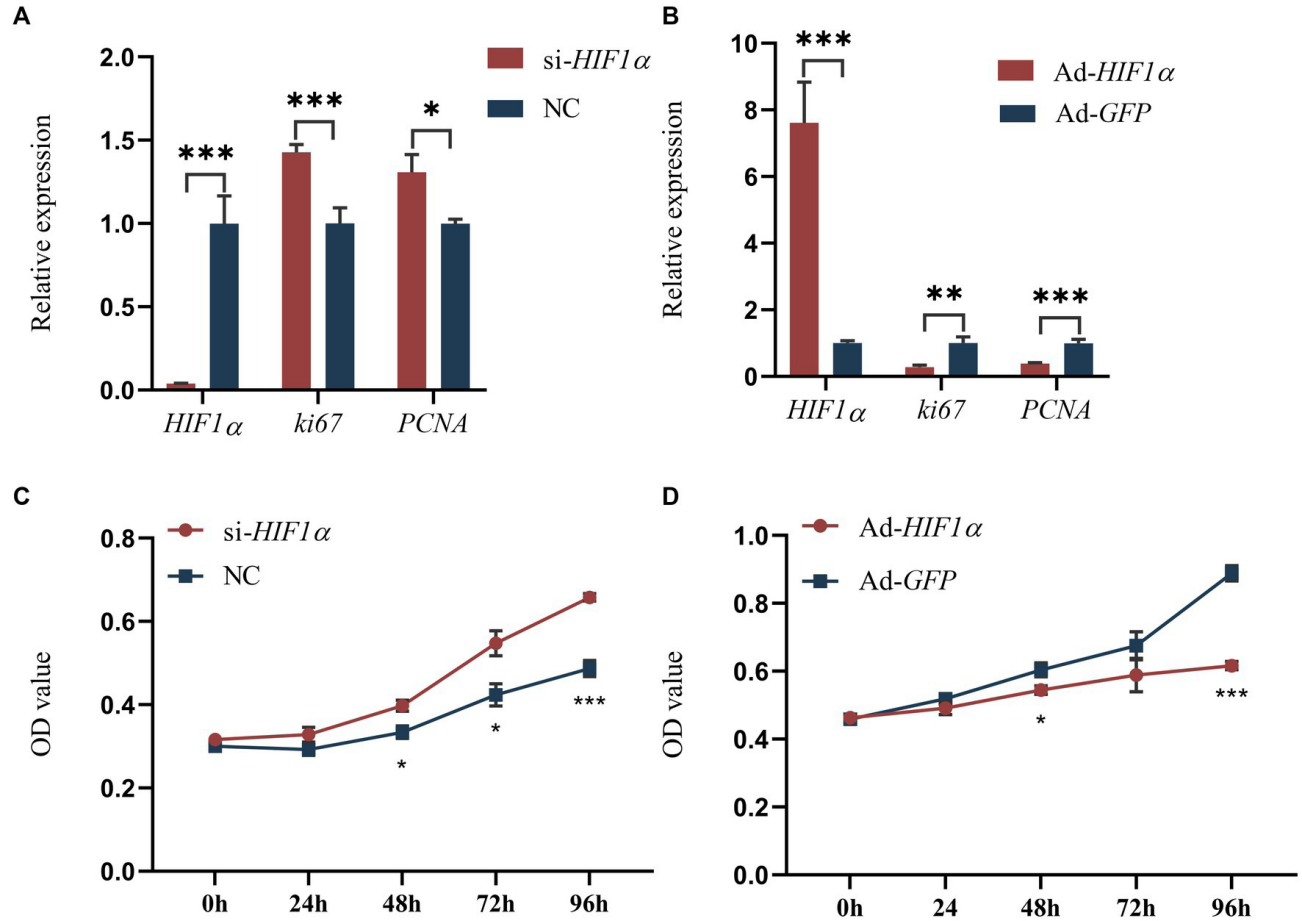
Impact of *HIF1α* on yak intramuscular preadipocyte proliferation. **(A,B)** Results from RT-qPCR. **(C,D)** CCK-8 outcomes post-transfection with si-*HIF1α* or Ad-*HIF1α* at intervals of 0, 24, 48, 72, and 96 h. Indicators *** signify *p* < 0.001, ** denote *p* < 0.01, and * represent *p* < 0.05.

Our sequencing results indicated that 109 target genes of DEMs were significantly enriched in the HIF-1 signaling pathway (ko04066), such as *HIF1α*, *IGF1*, *ARNT*, *PIK3CA*, *AKT3*, *STAT3* and *MAPK1* (Supplementary Table S11). It has been reported that the hypoxia-inducible factor (HIF1α) acts as a key factor in lipid metabolism (21). Consequently, we decided to investigate the effects of *HIF1α* on the proliferation and differentiation of yak intramuscular preadipocytes, employing adenovirus-mediated overexpression and siRNA-mediated interference techniques. Our RT-qPCR results (Figures 6A,B) indicated that the inhibition of *HIF1α* significantly reduced its mRNA level (*p* < 0.001). Conversely, the expression levels of proliferation-related genes *ki67* (*p* < 0.001) and *PCNA* (*p* < 0.05) were increased. However, an opposite trend was observed following the overexpression treatment (Figures 6A,B). We also utilized 200 μM Cobalt(II) chloride hexahydrate (22) to simulate a hypoxic environment and subsequently assessed cell proliferation. The results revealed that cell proliferation activity significantly increased after *HIF1α* inhibition and decreased following its overexpression (Figures 6C,D). These findings suggest that *HIF1α* may positively influence the proliferation of intramuscular preadipocytes in yaks.

**Figure 7:**
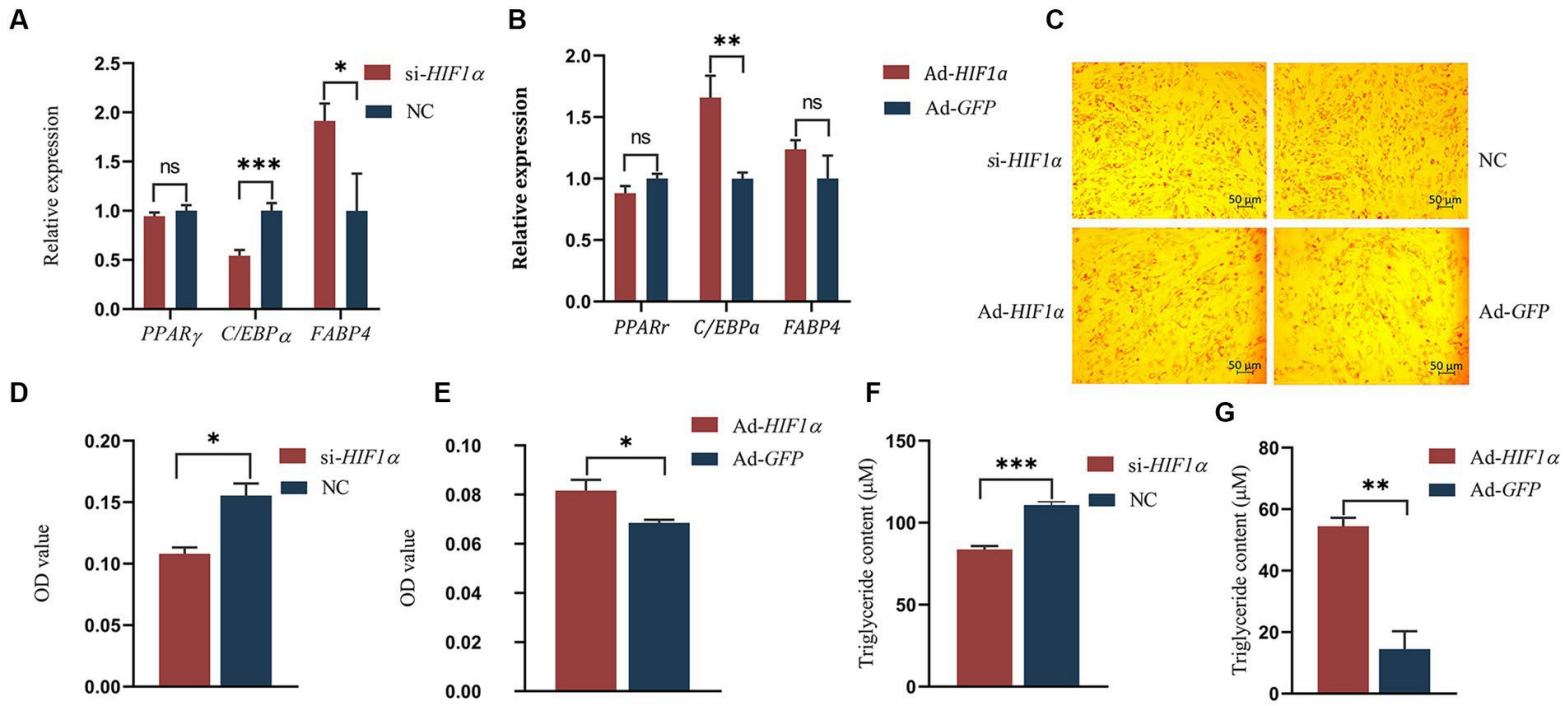
Impact of *HIF1α* on differentiation of yak intramuscular preadipocytes. **(A,B)** RT-qPCR results. **(C–E)** Oil Red O staining results post-transfection with si-*HIF1α* or Ad-*HIF1α* for 48 h. **(F,G)** TG test results. Indicators *** signify *p* < 0.001, ** denote *p* < 0.01, and * represent *p* < 0.05.

To evaluate the influence of *HIF1α* on the differentiation of intramuscular preadipocytes, we assessed the expression of differentiation-associated genes, namely *PPARγ* (23), *C/EBPα* (24), and *FABP4* (25), following either inhibition or overexpression of *HIF1α*. Our results revealed that the mRNA levels of *C/EBPα* were significantly downregulated (*p* < 0.01), while *FABP4* was notably enhanced (*p* < 0.05) following *HIF1α* knockdown compared to control cells (Figure 7A). Conversely, *HIF1α* overexpression significantly upregulated the expression of *C/EBPα* (*p* < 0.01) (Figure 7B). Under hypoxic conditions, lipid droplet formation was substantially reduced when *HIF1α* was inhibited (Figures 7C,D,F). In contrast, lipid droplet formation was significantly increased when *HIF1α* expression was enhanced (Figures 7C,E,G). These findings suggest that *HIF1α* might promote the differentiation of intramuscular preadipocytes in yaks.

## 4. Discussion

IMF, the fat accumulated between muscle fibers and within muscle cells, consists primarily of phospholipids and triglycerides. It is a crucial determinant of meat quality. Understanding the molecular mechanisms governing IMF deposition carries significant implications for animal husbandry. In this study, we initially identified 469 DEGs, 57 DEMs, and 73 DELs by sequencing the longissimus dorsi muscle of yaks with different IMF content. Subsequently, we predicted the targets of these DEMs and DELs. Remarkably, a substantial number of these DEGs, DEMs, and DELs were found to be enriched in pathways related to lipid metabolism and muscle development following functional annotation. Furthermore, many of these DEGs had known functions in lipid metabolism, such as *GLB1L2*, *APC2*, *H-FABP*, *PLPPR2*, and *ELOVL6*, and muscle development, including *METTL21C*, *MYL3*, and *MYO1G*. These genes likely represent some of the determinants of yak fat content, and their genetic functions within yaks are worth further investigation. The ceRNA hypothesis has garnered considerable attention in recent years. An increasing number of studies are endeavoring to construct ceRNA networks associated with non-coding RNAs during adipogenesis (26–28). In this study, we assembled a ceRNA network in yaks’ longissimus dorsi muscle, exhibiting varied IMF levels, and analyzed the potential adipogenic regulatory effects of these lncRNAs, miRNAs, and mRNAs. Overall, we constructed a ceRNA regulatory network encompassing 12 miRNAs, 11 lncRNAs, and 16 mRNAs. Within this network, it was apparent that four genes associated with lipid metabolism (*CREG1*, *ECHDC3*, *HMGCL*, and *FBXO17*) were targeted by miR-3059-x. Recent studies have reported that *CREG* heterozygous mice, when fed a high-fat diet, exhibited significant obesity and a 30% increase in body weight compared to their wild-type counterparts (29). Further, liver-specific knockout of *CREG*, coupled with a high-fat diet, led to liver steatosis, obesity, and insulin resistance (30). The Enoyl-CoA hydratase (ECH) is a pivotal mitochondrial enzyme involved in fatty acid β-oxidation, catalyzing the addition of H_2_O across the double bond of trans-2-enoyl-CoA, which forms a β-hydroxyacyl-CoA (31). Enoyl-CoA Hydratase Domain-Containing 3 (ECHDC3), a member of the ECH family, plays a regulatory role in adipocyte fatty acid metabolism, with *ECHDC3* silencing in human adipocytes significantly reducing insulin-stimulated glucose uptake and Akt Ser473 phosphorylation (32). The 3-hydroxy-3-methylglutaryl (HMG) CoA lyase (HMGCL) is a key enzyme in ketone body formation and is closely associated with energy metabolism (33). Meanwhile, the F-box protein (FBXO17) may inhibit the Wnt/β-catenin signaling pathway, thus influencing cancer progression (34). Our data demonstrated that these genes were differentially expressed in the longissimus dorsi muscle of yaks with differing fat content. Therefore, we hypothesize that the four identified genes (*CREG1*, *ECHDC3*, *HMGCL*, and *FBXO17*) and three new genes (*ENSBGRG00000010263*, *ENSBGRG00000000535*, and *ENSBGRG00000013053*) may play integral roles in regulating IMF deposition.

Furthermore, we identified several potential regulatory miRNAs (miR-1285-z, miR-122-y, miR-150-x, and miR-130-x). The principal biological function of miRNAs is carried out through pairing with a complementary site in the 3’UTR of their target mRNA, leading to post-transcriptional regulation by inhibiting translation. Most current research on miR-1285 concentrates on cancers, such as lung squamous cell carcinoma (35) and thyroid tumors (36). It has been found that TPI1, a pivotal enzyme in the glycolysis pathway, is a functional target of miR-1285-3p, and miR-1285-3p can diminish *TPI1* expression in Sertoli cells (37). Another study established that inhibiting miR-122 safeguards hepatocytes from lipid metabolic disorders such as NAFLD and curbs lipogenesis by elevating Sirt1 and activating the AMPK pathway (38). Moreover, mice with a knockout of miR-150 exhibited metabolic advantages including reduced body weight, decreased energy intake, and augmented lipid metabolism (39). In a study related to bovine adipogenesis, it was reported that bta-miR-130a/b could influence bovine adipocyte differentiation by targeting *PPARG* and Cytochrome P4502U1 (*CYP2U1*) (40). Interestingly, the miRNAs mentioned above showed significant differential expression between high-IMF and low-IMF samples in our study, suggesting that miR-1285-z, miR-122-y, miR-150-x, miR-130-x, and miR-3059-x may be implicated in the IMF deposition of yaks. In the ceRNA sub-networks we constructed, MSTRG.12131.3 and its target *ENSBGRG00000010263* communicated via the shared novel-m0258-5p and novel-m0259-5p response elements, whereas MSTRG.14096.1 and its target *ECHDC3*, and MSTRG.1417.5 and its target *CREG1* both communicated via miR-3059-x response elements. However, ENSBGRT00000028385 and its target *ENSBGRG00000000535*, and MSTRG.347.2 and its target *ENSBGRG00000013053* communicated via miR-10162-x and miR-1285-z response elements, respectively. Consequently, we hypothesize that these six sub-networks may play critical roles in the regulation of yak IMF deposition.

HIF1α, a transcription factor, is composed of a regulatory 120 kDa ɑ subunit and a 91–94 kDa β subunit, also known as ARNT (41). Under normoxic conditions, oxygen induces the hydroxylation of conserved proline residues (Pro402 and Pro564) in HIF1α subunits by oxygen-dependent HIF prolyl-4-hydroxylase domain enzymes (PHDs). This leads to the interaction with Von-Hippel-Lindau (VHL) protein, an ubiquitin ligase, and subsequent proteasomal degradation. Thus, HIF1α has a relatively short half-life of about 5–10 min under normal oxygen conditions (42, 43). However, under hypoxic conditions, cells lack sufficient O_2_, inhibiting HIF1α hydroxylation. This causes the HIF1α subunit to stabilize and accumulate in the cytoplasm. Subsequently, the accumulated HIF1α translocates to the nucleus, forming heterodimeric complexes with HIF-1β/ARNT (44, 45). This results in the recruitment and binding of p300/CBP to the hypoxia response element (HRE) in the promoter/enhancer region of the target gene, promoting its transcription. This process facilitates cellular response to hypoxia, crucial for maintaining oxygen homeostasis in the body (46–48). In recent years, the hypoxia adaptation mechanism of yaks, in which *HIF1α* plays a significant role, has garnered increased attention (49, 50). Moreover, several studies have indicated that *HIF1α* is implicated in hypoxia-induced lipid accumulation in hepatocytes (51), and is triggered by increased O_2_ consumption in adipocytes (52). Intriguingly, the expression level of *HIF1α* in yaks is significantly higher than that in cattle (53). *HIF1α* serves as a crucial gene in lipid metabolism and is also a vital player in the adaptation to hypoxic conditions. Given the yak’s remarkable ability to thrive in high-altitude, low-oxygen environments, we find the relationship between hypoxia-inducible factors like *HIF1α* and low-fat characteristics to be a compelling subject for additional investigation. While *HIF1α* is not part of our current ceRNA network study, it remains a focal point of our research agenda, warranting in-depth functional analysis. Moreover, our sequencing results revealed that 109 DEM targets were enriched in the HIF-1 signaling pathway (ko04066). Consequently, we investigated the regulatory effect of *HIF1α* on yak intramuscular preadipocytes. Cobalt chloride, a common model of chemical hypoxia (54), was employed. With the increase or inhibition of *HIF1α* expression, mRNA levels of *FABP4* and *C/EBPα*, excluding *PPARγ*, were notably upregulated or downregulated. Combining the results of triglyceride measurements, oil red O stain, and CCK-8 assays, we concluded that *HIF1α* negatively regulated the proliferation of yak intramuscular preadipocytes and positively influenced lipid droplet formation. These findings align with previous *HIF1α* studies in other species (55–57). Notably, *CREG* may activate *HIF1α* signaling via the inhibition of *VHL* expression (58). While its regulatory role in yak IMF deposition remains unclear, it provides a fresh perspective for future research.

## 5. Conclusion

Our study utilized high-throughput sequencing to analyze a large scale of differentially expressed mRNAs, miRNAs, and lncRNAs in longissimus dorsi muscle of yaks with high and low IMF content. Further, we constructed a ceRNA regulatory network based on the differentially expressed transcripts. Our findings revealed the genetic regulatory mechanisms and provided valuable data for understanding the low-fat traits of yaks. Additionally, we identified the regulatory role of *HIF1α* in lipid deposition and the proliferation of yak intramuscular preadipocytes. This discovery is particularly important for studying both the low-fat characteristics and the hypoxic adaptation of yaks.

## Data availability statement

The data supporting the conclusions of this article can be found in Supplementary Tables S1-14. The sequencing data were uploaded in the NCBI BioProject database with the accession number is PRJNA1014576, respectively.

## Ethics statement

The animal study was approved by the Institution Animal Care and Use Committee in the Southwest Minzu University. The study was conducted in accordance with the local legislation and institutional requirements.

## Author contributions

ML: Data curation, Formal analysis, Investigation, Methodology, Validation, Visualization, Writing – original draft, Writing – review & editing. HW: Conceptualization, Funding acquisition, Methodology, Resources. JZ: Funding acquisition, Resources, Writing – review & editing. KY: Funding acquisition, Resources, Writing – review & editing. SS: Funding acquisition, Resources, Writing – review & editing. CF: Funding acquisition, Resources, Writing – review & editing. JZ: Funding acquisition, Resources, Writing – review & editing. WP: Funding acquisition, Resources, Writing – review & editing.
