# Correction: IMF deposition ceRNA network analysis and functional study of HIF1a in yak

**DOI:** 10.3389/fvets.2026.1798878

**Published:** 2026-03-10

**Authors:** Mengning Luo, Hui Wang, Jun Zhang, Kangzhu Yixi, Shi Shu, Changqi Fu, Jincheng Zhong, Wei Peng

**Affiliations:** 1 Key Laboratory of Qinghai-Tibetan Plateau Animal Genetic Resource Reservation and Utilization, Sichuan Province and Ministry of Education, Southwest Minzu University, Chengdu, China; 2 Qinghai Academy of Animal Science and Veterinary Medicine, Qinghai University, Xining, China

**Keywords:** IMF, adipogenesis, RNA-seq, *HIF1*α, ceRNA, yak

The **Data availability statement** was erroneously given as “The datasets presented in this study can be found in online repositories. The names of the repository/repositories and accession number(s) can be found in the article/supplementary material.”.

The correct **Data availability statement** is:

“The data supporting the conclusions of this article can be found in Table S1 to Table 14. The sequencing data were uploaded in the NCBI BioProject database with the accession number is PRJNA1014576, respectively.”

Supplemental Data Sheet 1 was erroneously published with the original version of this paper. The file has now been replaced with **Supplementary Tables 1–14**.

A correction has been made to the **Ethics statement**.

The **Ethics statement** was erroneously given as “The part of the study involving animal samples was reviewed and approved by the Institution Animal Care and Use Committee in the Southwest Minzu University (Permit number: SMU 202106010 revised in 1 June 2021), Chengdu, China. The animal study was approved by the Institution Animal Care and Use Committee in the Southwest Minzu University. The study was conducted in accordance with the local legislation and institutional requirements.”

The correct ethics statement is: “The animal study was approved by the Institution Animal Care and Use Committee in the Southwest Minzu University. The study was conducted in accordance with the local legislation and institutional requirements.”

The original version of this article has been updated.

